# Occurrence of pseudoaneurysm of the coronary button due to aortic remodeling after Bentall operation

**DOI:** 10.1093/jscr/rjae080

**Published:** 2024-02-22

**Authors:** Takeyuki Kanemura, Yoshinori Nakahara, Retsu Tateishi, Fumiya Haba, Shunya Ono

**Affiliations:** Department of Cardiovascular Surgery, IMS Katsushika Heart Center, 3-30-1 Horikiri, Katsushika Ward, Tokyo 124-0006, Japan; Department of Cardiovascular Surgery, IMS Katsushika Heart Center, 3-30-1 Horikiri, Katsushika Ward, Tokyo 124-0006, Japan; Department of Cardiovascular Surgery, IMS Katsushika Heart Center, 3-30-1 Horikiri, Katsushika Ward, Tokyo 124-0006, Japan; Department of Cardiovascular Surgery, IMS Katsushika Heart Center, 3-30-1 Horikiri, Katsushika Ward, Tokyo 124-0006, Japan; Department of Cardiovascular Surgery, IMS Katsushika Heart Center, 3-30-1 Horikiri, Katsushika Ward, Tokyo 124-0006, Japan

**Keywords:** Bentall procedure, pseudoaneurysm, aortic dissection

## Abstract

Here, we present a case report detailing a pseudoaneurysm of the coronary button due to aortic remodeling that occurred 2 years after aortic root replacement. The patient was referred to our hospital with a diagnosis of left coronary artery pseudoaneurysm. Intraoperative findings revealed substantially loosened sutures in both the left and right coronary arteries with bleeding. Specifically, the left coronary artery was detached at the 6–9 o’clock positions. The operation was concluded with ligation of the loose suture and addition of a new suture. Chronic dissection thickened the aortic wall of the coronary artery ostium in the initial Bentall operation, whereas the sutured coronary button in this operation exhibited a normal arterial wall without a thickened dissected intima. This suggests that aortic wall remodelling of the coronary ostium leads to suture loosening and subsequent haemorrhage. Aortic wall remodeling may lead to bleeding or pseudoaneurysms during the remote period.

## Introduction

Although the original technique has a high incidence of coronary button complications, the Bentall procedure remains the gold standard for aortic root replacement. Modifications have been proposed to address this problem [1, 2] to the Society of Thoracic Surgeons database, between 2000 and 2011, valved conduit replacement resulted in early mortality rates of 8.9% for adult patients undergoing radical reconstruction. However, a systematic review reported a 10-year cumulative incidence of 14.1% for major bleeding and thromboembolic complications after Bentall surgery [3]. Post-Bentall pseudoaneurysms have been reported in 6–10% of patients [4, 5]. Our case report describes a pseudoaneurysm of the coronary button 2 years after aortic root replacement due to remote remodeling of the aortic wall of the coronary ostium.

## Case report

**Figure 1:**
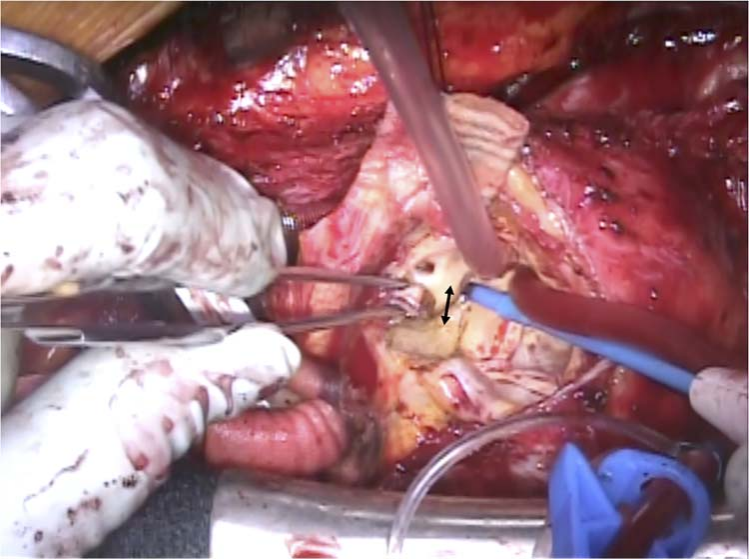
Intraoperative view of previous Bentall procedure. The arrow indicates the thickened aortic wall of the left coronary ostium due to the chronic dissection.

**Figure 2:**
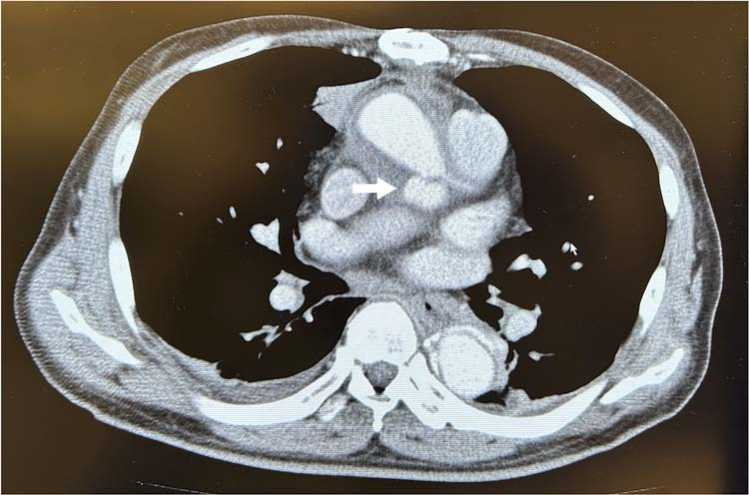
Computed tomography scans revealed a pseudoaneurysm from the left coronary artery. The arrow indicates the pseudoaneurysm.

A 67-year-old male patient underwent emergency total arch replacement 11 years prior to acute aortic dissection. Nine years later, the patient underwent descending aortic replacement surgery because of enlargement of the descending aorta. Two years later, the Bentall procedure was performed for chronic dissection of the aortic root and subsequent enlargement. We identified the dissected aortic wall of the coronary ostium, which was thickened (Fig. 1) and created a coronary button for anastomosis. The patient presented with haemoptysis was diagnosed with a pseudoaneurysm in the left coronary artery using computed tomographic angiography (Fig. 2), and was referred to our hospital for surgery.

**Figure 3:**
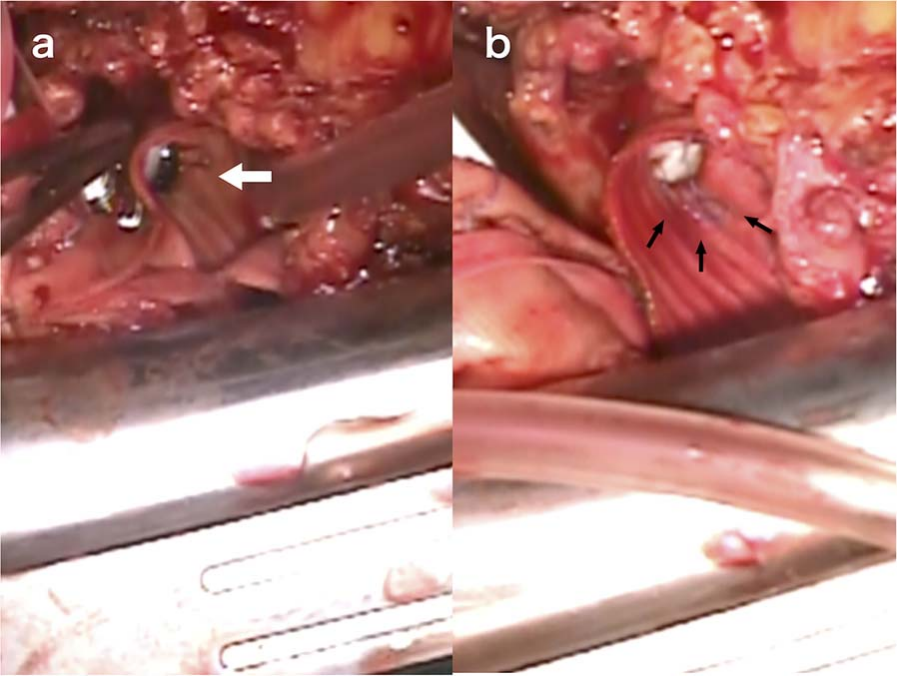
Intraoperative view of reoperation. The arrow indicates the dehiscence in the 6–9 o’clock thread of the left coronary button (a). The arrow indicates the loose thread (b).

During reoperation, the right femoral artery and vein were cannulated to institute the cardiopulmonary bypass and cool the patient down to 28°C. After re-sternotomy, bleeding was observed in both coronary buttons, with a large haematoma surrounding the artificial vessels. The ascending aortic graft was cross-clamped and myocardial protection was achieved through antegrade cardioplegia. After transverse incision of the artificial vessel, we observed remodeling of the aortic walls of the left and right coronary buttons, which had thinned since the previous surgery. Dehiscence was observed in the 6–9 o’clock thread of the left coronary button with no cutting of the aortic wall (Fig. 3a). Both the left and right coronary artery threads were loose (Fig. 3b), necessitating ligation with prior and additional sutures. The transverse incision of the artificial graft was closed, and the aortic clamp was released. The patient was extubated on the day after surgery and discharged on the 10th postoperative day.

## Discussion

We present a case of a pseudoaneurysm of the right and left coronary ostium 2 years after a Bentall operation for chronic aortic root dissection. Aortic remodeling led to loosening of the coronary button sutures, resulting in a pseudoaneurysm. Retrospective analysis revealed thickening of the aortic wall of the coronary button due to chronic dissection during the initial surgery, whereas no thickening was observed during the secondary surgery. The patient did not present with connective tissue disorders, inflammatory diseases, or infectious complications. These findings suggest that the pseudoaneurysm resulted from remodeling of the aortic wall thickened by aortic dissection, which led to suture loosening. Although severe calcification and fragility of the aortic wall, immature surgery, infection, and connective tissue diseases such as Marfan syndrome [6–9] have been reported as causes of pseudoaneurysms after Bentall’s procedure, no reports have referred to the remodeling of the aortic wall.

To enhance the outcomes after aortic root replacement, it is imperative to focus on remodeling as a cause of pseudoaneurysms. Aortic remodeling is a well-known phenomenon following entry resection for aortic dissection with interventions such as thoracic endovascular aortic repair [10–12]. Rapid aortic remodeling has also been observed after thoracic endovascular aortic repair for retrograde type A aortic dissection [12]. Furthermore, it is not surprising that this remodeling also occurs in coronary buttons with chronic dissection after entry resection. For coronary ostium anastomosis, where the proportion of aortic wall thickness in the total sutures used is high, even minimal loosening from chronic dissection remodelling can lead to haemorrhage. To prevent pseudoaneurysms resulting from remodeling, coronary artery reconstruction should involve trimming the thickened aortic wall caused by chronic dissection and minimizing the residual wall thickness.

In conclusion, aortic wall remodeling of the coronary ostium after aortic root replacement for chronic dissection may lead to bleeding or pseudoaneurysms during the remote period.
